# In-silico identification and comparison of transcription factor binding sites cluster in anterior-posterior patterning genes in *Drosophila melanogaster* and *Tribolium castaneum*

**DOI:** 10.1371/journal.pone.0290035

**Published:** 2023-08-17

**Authors:** Anshika Moudgil, Ranbir Chander Sobti, Tejinder Kaur

**Affiliations:** 1 Department of Zoology, DAV University, Jalandhar, Punjab, India; 2 Department of Biotechnology, Panjab University, Chandigarh, India; University of Delhi, INDIA

## Abstract

The cis-regulatory data that help in transcriptional regulation is arranged into modular pieces of a few hundred base pairs called CRMs (cis-regulatory modules) and numerous binding sites for multiple transcription factors are prominent characteristics of these cis-regulatory modules. The present study was designed to localize transcription factor binding site (TFBS) clusters on twelve Anterior-posterior (A-P) genes in *Tribolium castaneum* and compare them to their orthologous gene enhancers in *Drosophila melanogaster*. Out of the twelve A-P patterning genes, six were gap genes (*Kruppel*, *Knirps*, *Tailless*, *Hunchback*, *Giant*, and *Caudal*) and six were pair rule genes (*Hairy*, *Runt*, *Even-skipped*, *Fushi-tarazu*, *Paired*, and *Odd-skipped*). The genes along with 20 kb upstream and downstream regions were scanned for TFBS clusters using the Motif Cluster Alignment Search Tool (MCAST), a bioinformatics tool that looks for set of nucleotide sequences for statistically significant clusters of non-overlapping occurrence of a given set of motifs. The motifs used in the current study were Hunchback, Caudal, Giant, Kruppel, Knirps, and Even-skipped. The results of the MCAST analysis revealed the maximum number of TFBS for Hunchback, Knirps, Caudal, and Kruppel in both *D*. *melanogaster* and *T*. *castaneum*, while Bicoid TFBS clusters were found only in *D*. *melanogaster*. The size of all the predicted TFBS clusters was less than 1kb in both insect species. These sequences revealed more transversional sites (Tv) than transitional sites (Ti) and the average Ti/Tv ratio was 0.75.

## Introduction

**Fig 1:**
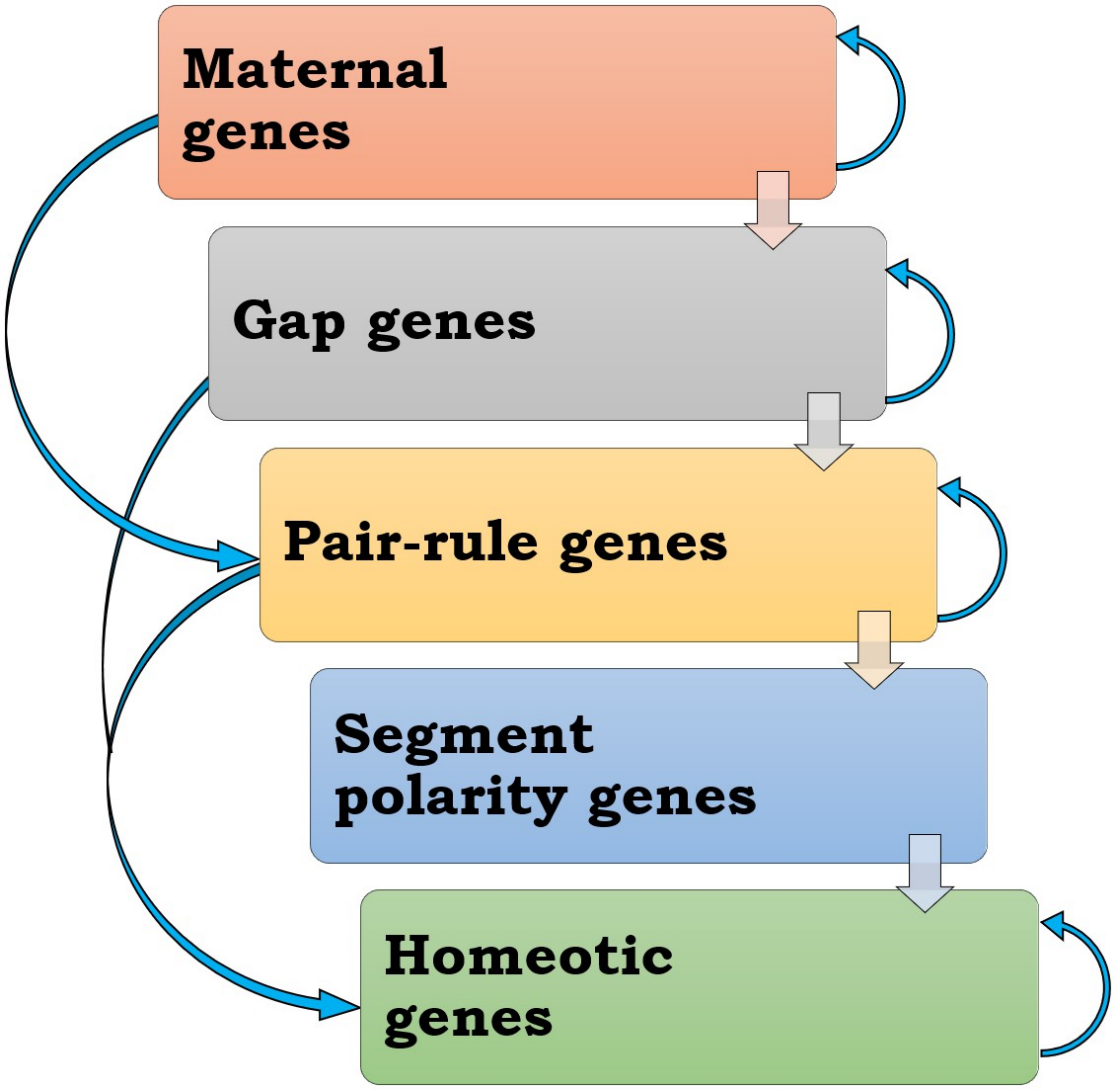
Schematic representation of the regulatory relationship between the anterior-posterior patterning gene cascade in *Drosophila melanogaster*.

To know the development processes occurring in metazoans, it is vital to comprehend the regulatory mechanics of the underlying transcriptional network. The genomic sequence of an organism contains a significant amount of information that specifies how and when genes will be expressed. Despite the availability of genome sequences for many metazoans, very little is known about how this biological data is encoded [1, 2]. Previous research on the early development of *Drosophila melanogaster*, a model organism for more than three decades, provides an excellent context for studying the cis-regulatory modules (CRMs). CRMs are certain areas of non-protein-coding DNA, that play a significant role in controlling the expression patterns of genes to build an embryo’s tissue [3]. The CRMs are composed of groups of short DNA sequences that are acknowledged and bound by certain transcription factors [4]. The enhancers, promoters, and silencers consist of cis-regulatory sequences, recognized as CRMs [5]. Enhancers and silencers are usually found upstream (5′), downstream (3′), or in the intron (or introns) of the gene they control, although they can also be found far away while promoter sequences are always present upstream to the target gene [6]. During the early stages of embryonic development, a quick cascade of gene regulation determines the segmented body pattern of *D*. *melanogaster* [7]. The process of Anterior-Posterior (A-P) segmentation is initiated by the maternal gene products that are present in the gradient. The bicoid protein gradient exhibiting morphogenetic features is the best-known example. A spatial-differential, concentration-dependent expression (or repression), of certain zygotic genes, is regarded to establish the bicoid protein gradient [8, 9]. The first zygotic gene to be expressed belongs to the gap genes cascade which is a well-studied system in *D*. *melanogaster* [10]. The gap genes control the pair-rule genes which further regulate the segment polarity genes and the Homeotic gene complex in succession [10–13]. In addition to this, the maternal genes, gap genes, pair-rule genes, and Homeotic gene complex also self-regulate themselves as shown in Fig 1. An ideal framework for researching the cis-regulatory sequences is provided by all research on the early stages of *Drosophila* development. A variety of interactions between different transcription factors (TF) and their target regulatory areas have been thoroughly defined [11, 14], and comparative investigations have demonstrated that cis-regulatory regions are often functionally conserved throughout the genus [15–17]. Previous research characterized transcription factor binding site (TFBS) locations in the early *Drosophila* embryo and estimated the binding affinity of each factor using Position weight matrices (PWMs) [1, 18]. PWMs are a valuable method for analysing the location of potential binding sites and estimating their binding strength [19, 20]. All the research about the cis-regulatory modules is mainly focused on the *Drosophila* genus. Not much has been done on other insect species. Over the last two decades, *T*. *castaneum* has emerged as a potent organism to study short germ segmentation, embryonic head and leg development, metamorphosis, and in insect biology. The anterior-posterior patterning in *Tribolium* follows an ancestral route i.e. short germ embryogenesis, which is different from *D*. *melanogaster* which follows the latest route i.e. long germ embryogenesis [21, 22]. In this paper, we investigate the Transcription factor binding sites cluster i.e. Cis-regulatory modules in the gap genes and pair-rule genes of *D*. *melanogaster*, and compare them with their orthologs present in *T*. *castaneum*.

## Material and methods

### Data collection

The Translated gene sequences for the A-P patterning genes of *D*. *melanogaster* were downloaded from Flybase (FB2021_03) [23]. The NCBI database (pBlast) was then used to collect the A-P patterning protein sequences of *T*. *castaneum* orthologous to those of *D*. *melanogaster* [24]. The orthologous sequences with a high query-covered value and a low error value were selected. The genomic data viewer was used to access these sequences. Following that, 20 kb flanking sequences were added both upstream (-) and downstream (+) to each target gene’s sequence. The gene sequences along with the additional flanking sequences were then downloaded in the FASTA format. The sequence location of genes alongwith the flanking regions are given in Tables 1 and 2 for *D*. *melanogaster* and *T*. *castaneum* respectively.

**Table 1:** List of genes used for enhancer localization along with their chromosomal locations, accession numbers, gene locations, and gene sequence location with the flanking regions in *Drosophila melanogaster*.

S.No.	Gene Name	Gene chromosomal location and Accession number	Gene Location	Gene sequences along with their 20 kb flanking regions
*1*	*Hunchback*	Chr. 3R (NT_033777.3)	8691649–8694381	8671649–8714381
*2*	*Knirps*	Chr. 3L (NT_037436.4)	20692673–20694617	20672673–20714617
*3*	*Caudal*	Chr. 2L (NT_033779.5)	20769990–20783396	20749990–20803396
*4*	*Kruppel*	Chr. 2R (NT_033778.4)	25226133–25231872	25206133–25251872
*5*	*Giant*	Chr. X (NC_004354.4)	24272888–2428993	2407288–2448993
*6*	*Tailless*	Chr. 3R(NT_033777.3)	30852398–30854173	30832398–30874173
*7*	*Even-skipped*	Chr. 2R (NT_033778.4)	9979170–9980942	9959170–10000942
*8*	*Odd-skipped*	Chr. 2L (NT_033779.5)	3604856–3606494	3584856–3626494
*9*	*Runt*	Chr. X (NC_004354.4)	20690004–20697985	20670004–20717985
*10*	*Hairy*	Chr. 3L (NT_037436.4)	8676032–8678639	8656032–8698639
*11*	*Fushi-tarazu*	Chr. 3 (NT_033777.3)	6864,255–6865910	6844,255–6885,910
*12*	*Paired*	Chr. 2L (NT_033779.5)	12083091–12085750	12063091–12105750

**Table 2:** List of genes used for enhancer localization along with their chromosomal locations, accession numbers, gene locations, and gene sequence location with the flanking regions in *Tribolium castaneum*.

S.No.	Gene Name	Gene chromosomal location and Accession number	Gene Location	Gene sequences along with their 20 kb flanking regions
*1*	*Hunchback*	Chr. LG5 (NC_007420.3)	8101789–8103523	8081615–8123697
*2*	*Knirps*	Chr. LG3 (NC_007418.3)	4699064–4709975	4679064–4729975
*3*	*Caudal*	Chr. LG4 (NM_007419.2)	2828167–2845622	2808167–2865622
*4*	*Kruppel*	Chr. LG10 (NC_007425.3)	6495006–6502111	6475006–6522111
*5*	*Giant*	Chr. LG4 (NC_007419.2)	3765785–3766872	3745785–3786872
*6*	*Tailless*	Chr. LG2 (NC_007417.3)	11221687–11225898	11201687–11245898
*7*	*Even-skipped*	Chr. LG7 (NC_007422.5)	624611–628174	604611–648174
*8*	*Odd-skipped*	Chr. LG8 (NC_007423.3)	7672873–7679594	7652873–7699594
*9*	*Runt*	Chr. LG8 (NC_007423.3)	11253714–11257683	11233714–11277683
*10*	*Hairy*	Chr. LG9(NC_007424.3)	15627964–15629572	15607964–15649572
*11*	*Fushi-tarazu*	Chr. LG2 (NC_007417.3)	8414592–8415698	8394592–8435698
*12*	*Paired*	Chr. LG6 (NC_007421.3)	8595852–8650582	8575852–8650582

### Motif collection

The JASPAR database, which is open to the public, contains position weight matrices (PWMs), of various species in six taxonomic groupings. The PWM motifs used for the present study were Bicoid, Hunchback, Caudal, Giant, Kruppel, Knirps, and Even-skipped of *D*. *melanogaster*. These were downloaded in meme format from JASPAR software [25].

### Meme suite analysis

For the discovery of transcription factor binding site (TFBS) clusters, MCAST, an application of MEME Suite was run [26]. MCAST scans for clusters of matches to one or more nucleotide motifs in sequences [27]. The input of sequence was given in text file in FASTA format while the motifs were given in Meme format. ARR1 of *Saccharomyces cerevisiae* was used as an outgroup. For the identification of TFBS, the parameters which were used in the present study were: p-value should be less than 0.005, the error value less than 5 and the gap between two TFBS should be less than 30 base pairs. The result was displayed in the HTML format and a cluster with greater motif score, and low error value was selected as pCRM.

### Annotation of transcription start site (TSS)

Transcription start sites (TSS) for the A-P genes were predicted using the genome data viewer tool of NCBI in the case of *D*. *melanogaster* and *T*. *castaneum* [28].

### Annotation of promotor region, exon, intron region of the gene

Putative promoter regions, exon, and intronic regions were identified using two databases Ensemble and NCBI [28, 29].

The NCBI database’s genome data viewer was used to get the necessary sequence, which featured intron, exon, and promoter sequences. The intron and exon of the given sequence were represented by different colours. Exons were represented by light pink, introns by green, and the following gene by blue colour. The promotor sequence area was recovered up to 1000 bp upstream of the TSS region, including the AT-rich region and the TATA box.

The searched-for sequence, which contained the 5’ flanking area, the promotor, the exon, the intron, and the 3’ flanking region, was retrieved from the Ensemble website.

### Sequence alignment and variation of predicted cis-regulatory modules

All the predicted pCRM were aligned using the ClustalW [30] tool in Bioedit software [31] and these aligned sequences were subjected to calculate the conserved sites, transition pairs (Ti), transversional pairs (Tv), and transition/transversion (Ti/Tv) ratio in MEGA XI software [32].

### Interaction between the TFs

In the present study, the STRING database was used to predict the protein-protein interactions between the different transcription factors searched for their binding sites on the A-P patterning genes [33].

## Results

### TFBS clusters in gap genes of *D*. *melanogaster* and *T*. *castaneum*

**Fig 2:**
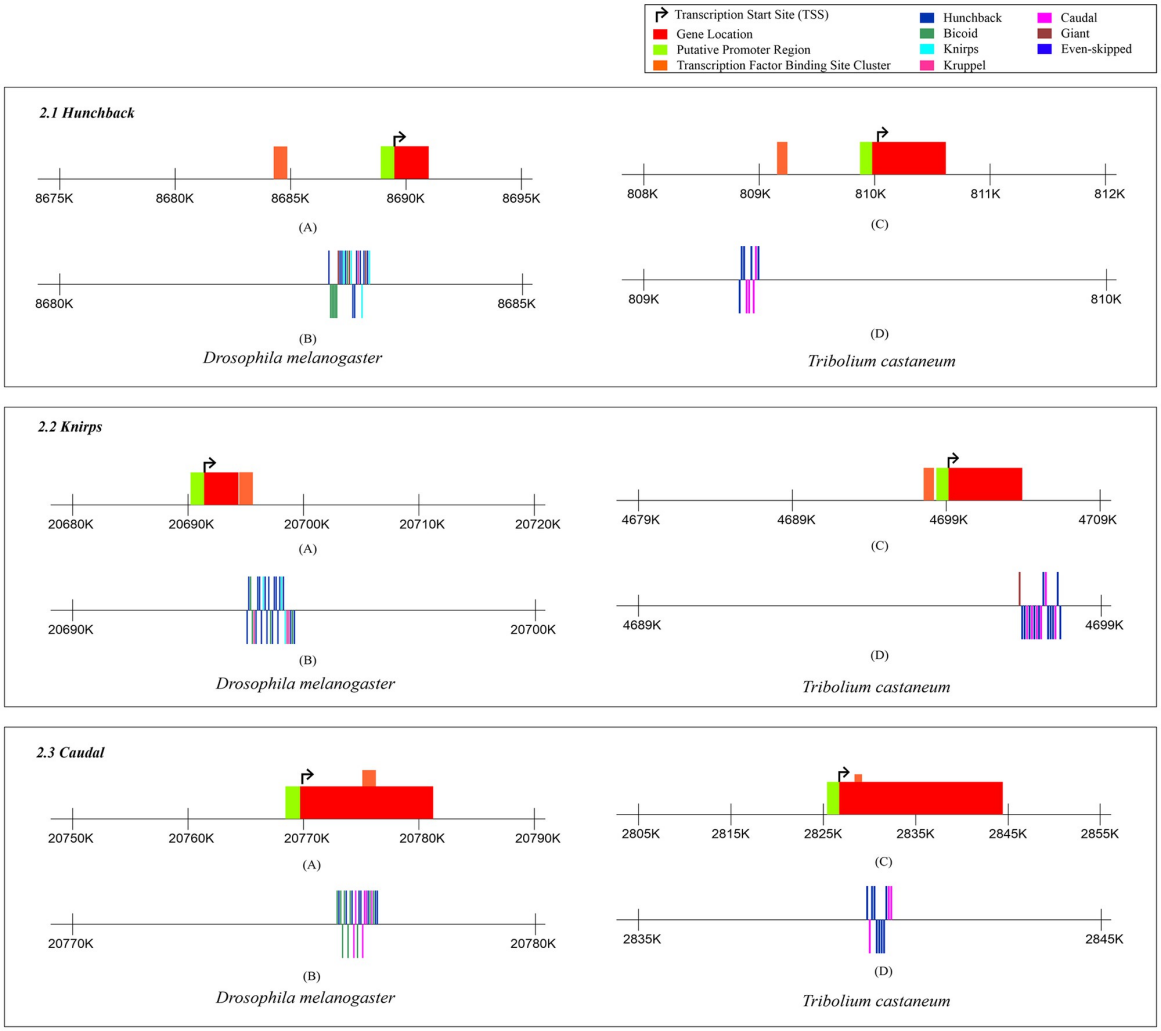
Figure showing the pCRM details of *Drosophila melanogaster* and *Tribolium castaneum* for different transcription factors in *Hunchback*, *Knirps*, and *Caudal*. 2.1 shows the predicted transcription factor binding site clusters in *Hunchback* for *D*. *melanogaster* and *T*. *castaneum*.A) Locations of the gene, transcription start site (TSS) and the transcription factor binding site (TFBS) cluster as predicted by the MCAST software for the *Hunchback* gene in *D*. *melanogaster*, B) Binding sites for different transcription factors on the predicted TFBS cluster in the gene *Hunchback* for *D*. *melanogaster*, C) Locations of the gene, TSS and the TFBS cluster as predicted by the MCAST software for the *Hunchback* gene in *T*. *castaneum*, D) Binding sites for different transcription factors on the predicted TFBS cluster in the gene *Hunchback* for *T*. *castaneum*. 2.2 shows the predicted transcription factor binding site clusters in *Knirps* for *D*. *melanogaster* and *T*. *castaneum*. A) Locations of the gene, TSS and the TFBS cluster as predicted by the MCAST software for the *Knirps* gene in *D*. *melanogaster*, B) Binding sites for different transcription factors on the predicted TFBS cluster in the gene *Knirps* for *D*. *melanogaster*, C) Locations of the gene, TSS and the TFBS cluster as predicted by the MCAST software for the *Knirps* gene in *T*. *castaneum*, D) Binding sites for different transcription factors on the predicted TFBS cluster in the gene *Knirps* for *T*. *castaneum*, 2.3 shows the predicted transcription factor binding site clusters in *Caudal* for *D*. *melanogaster* and *T*. *castaneum*. A) Locations of the gene, TSS and the TFBS cluster as predicted by the MCAST software for the *Caudal* gene in *D*. *melanogaster*, B) Binding sites for different transcription factors on the predicted TFBS cluster in the gene *Caudal* for *D*. *melanogaster*, C) Locations of the gene, TSS and the TFBS cluster as predicted by the MCAST software for the *Caudal* gene in *T*. *castaneum*, D) Binding sites for different transcription factors on the predicted TFBS cluster in the gene *Caudal* for *T*. *castaneum*.

The results of the MCAST analysis for identifying TFBS clusters in the gap genes (*Hunchback*, *Knirps*, *and Caudal)* are depicted in Fig 2. The results reveal that the location of transcription factor binding sites (TFBS) on the *Hunchback* gene is upstream to the transcription start site (TSS) in both *D*. *melanogaster* and *T*. *castaneum* (Fig 2.1). However, the results are variable in *D*. *melanogaster* and *T*. *castaneum* for another gap gene called *Knirps*, on which, the TFBS cluster is located within intron 2 of the gene in the dipteran insect and upstream in the coleopteron (Fig 2.2). As far as the *Caudal* gene is concerned, the cluster of TFBS is located downstream to the TSS in both the insect species (Fig 2.3). The cluster was found to be present within exon 1 of the gene in *D*. *melanogaster* and exon 3 of the gene in *T*. *castaneum*.

**Fig 3:**
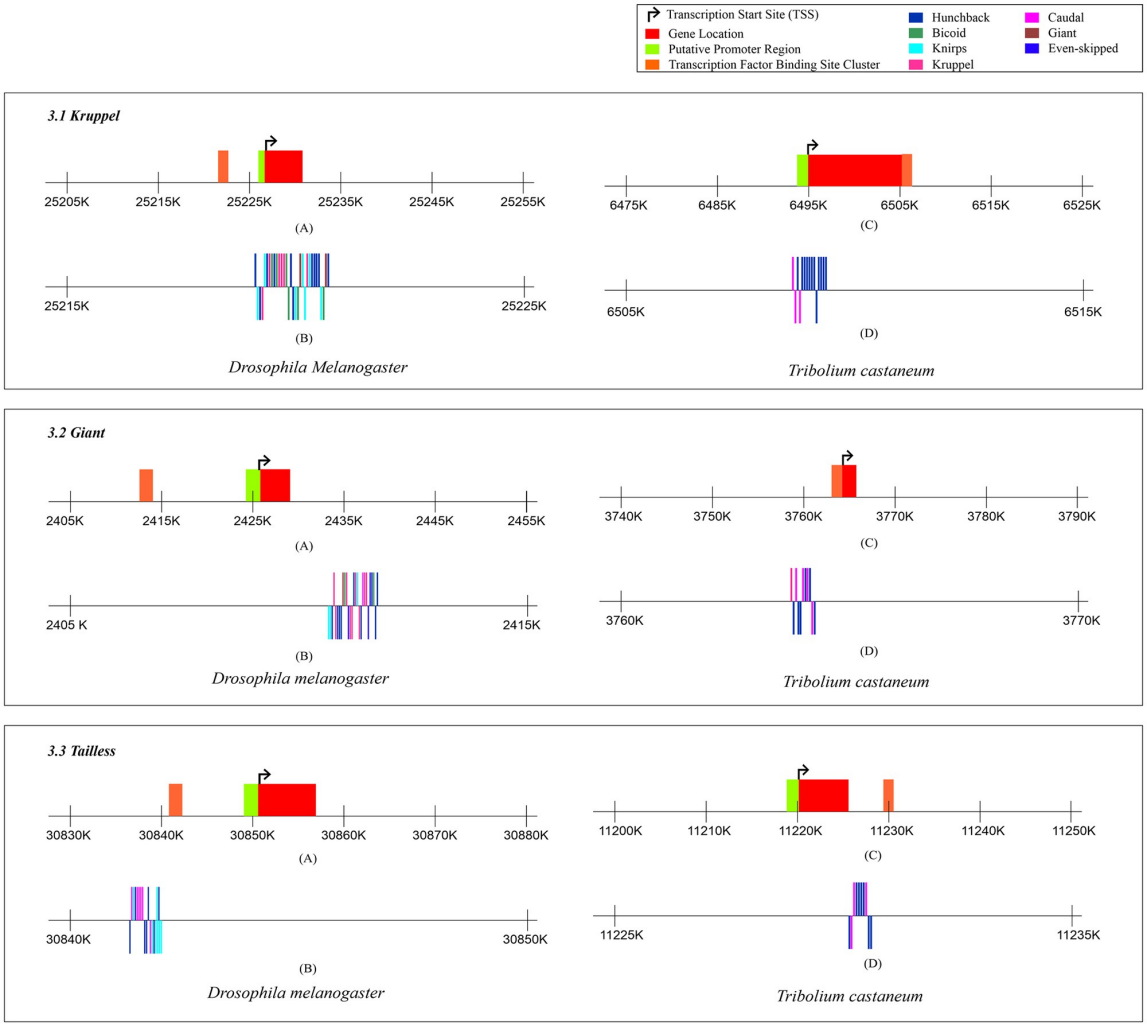
Figure showing the pCRM details of *Drosophila melanogaster* and *Tribolium castaneum* for different transcription factors in *Kruppel*, *Giant*, and *tailless*. 3.1 shows the predicted transcription factor binding site clusters in *Kruppel* for *D*. *melanogaster* and *T*. *castaneum*. A) Locations of the gene, TSS and the TFBS cluster as predicted by the MCAST software for the *Kruppel* gene in *D*. *melanogaster*, B) Binding sites for different transcription factors on the predicted TFBS cluster in the gene *Kruppel* for *D*. *melanogaster*, C) Locations of the gene, TSS and the TFBS cluster as predicted by the MCAST software for the *Kruppel* gene in *T*. *castaneum*, D) Binding sites for different transcription factors on the predicted TFBS cluster in the gene *Kruppel* for *T*. *castaneum*, 3.2 shows the predicted transcription factor binding site clusters in *Giant* for *D*. *melanogaster* and *T*. *castaneum*. A) Locations of the gene, TSS and the TFBS cluster as predicted by the MCAST software for the *Giant* gene in *D*. *melanogaster*, B) Binding sites for different transcription factors on the predicted TFBS cluster in the gene *Giant* for *D*. *melanogaster*, C) Locations of the gene, TSS and the TFBS cluster as predicted by the MCAST software for the *Giant* gene in *T*. *castaneum*, D) Binding sites for different transcription factors on the predicted TFBS cluster in the gene *Giant* for *T*. *castaneum* 3.3 shows the predicted transcription factor binding site clusters in *Tailless* for *D*. *melanogaster* and *T*. *castaneum*. A) Locations of the gene, TSS and the TFBS cluster as predicted by the MCAST software for the *Tailless* gene in *D*. *melanogaster*, B) Binding sites for different transcription factors on the predicted TFBS cluster in the gene *Tailless* for *D*. *melanogaster*, C) Locations of the gene, TSS and the TFBS cluster as predicted by the MCAST software for the *Tailless* gene in *T*. *castaneum*, D) Binding sites for different transcription factors on the predicted TFBS cluster in the gene *Tailless* for *T*. *castaneum*.

Fig 3 illustrates the results of MCAST analysis for predicting TFBS clusters on *Kruppel*, *Giant*, and *Tailless* genes. The TFBS cluster in the *Kruppel* gene is present upstream to the TSS in *D*. *melanogaster* and downstream to TSS in *T*. *castaneum* (Fig 3.1). In case of the *Giant* gene, the cluster of TFBS is present upstream to TSS in both *D*. *melanogaster* and *T*. *castaneum* as depicted in Fig 3.2. The location of the TFBS cluster in the gene *Tailless* is upstream in *D*. *melanogaster* while downstream to the TSS in *T*. *castaneum*.

### TFBS clusters in Pair rule genes of *D*. *melanogaster* and *T*. *castaneum*

**Fig 4:**
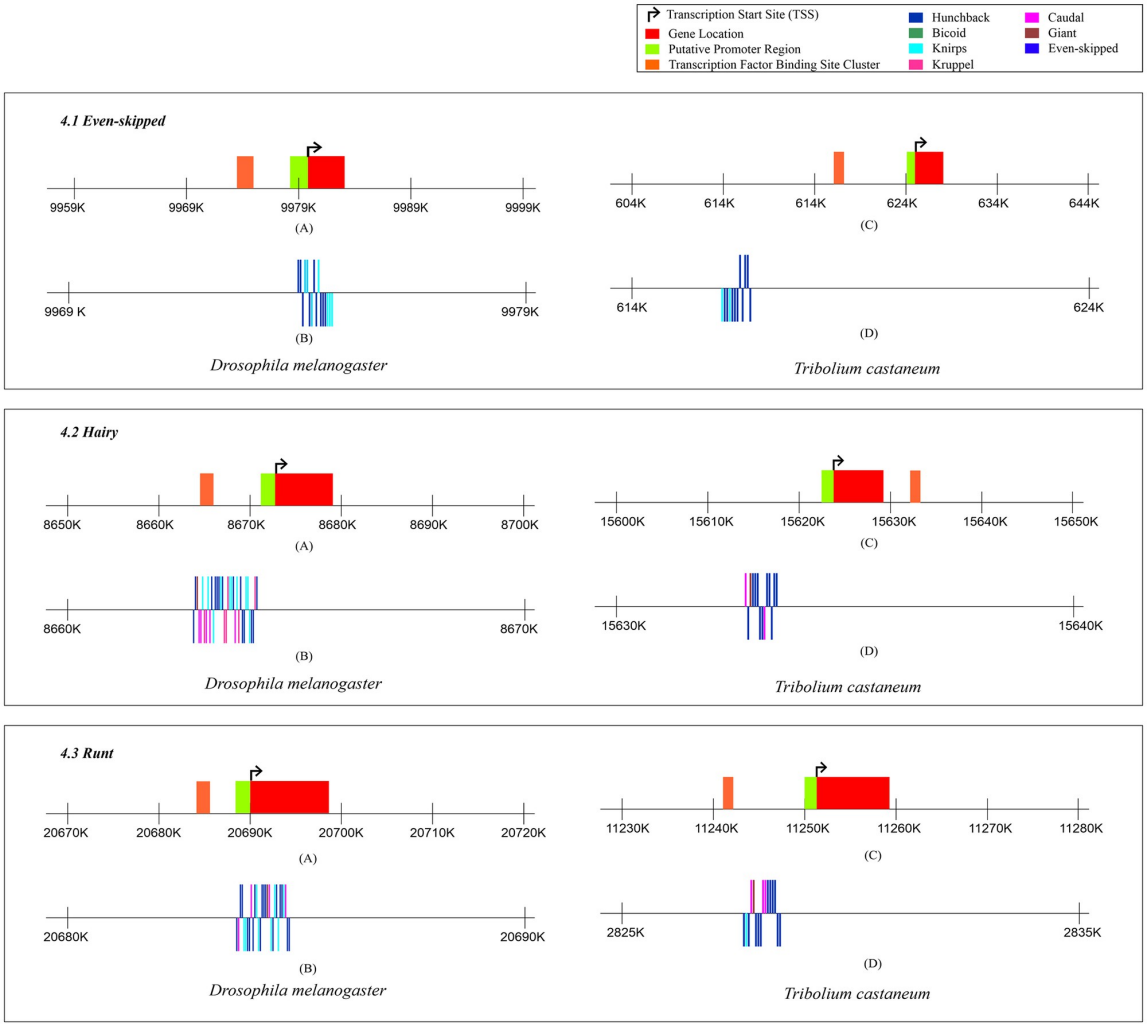
Figure showing the pCRM for *Drosophila melanogaster* and *Tribolium castaneum* for different transcription factors in *Even-skipped*, *Hairy*, and *Runt*. 4.1 shows the predicted transcription factor binding site clusters in *Even-skipped* for *D*. *melanogaster* and *T*. *castaneum*, A) Locations of the gene, TSS and the TFBS cluster as predicted by the MCAST software for the *Even-skipped* gene in *D*. *melanogaster*, B) Binding sites for different transcription factors on the predicted TFBS cluster in the gene *Even-skipped* for *D*. *melanogaster*, C) Locations of the gene, TSS and the TFBS cluster as predicted by the MCAST software for the *Even-skipped* gene in *T*. *castaneum*, D) Binding sites for different transcription factors on the predicted TFBS cluster in the gene *Even-skipped* for *T*. *castaneum*. 4.2 shows the predicted transcription factor binding site clusters in *Hairy* for *D*. *melanogaster* and *T*. *castaneum*. A) Locations of the gene, TSS and the TFBS cluster as predicted by the MCAST software for the *Hairy* gene in *D*. *melanogaster*, B) Binding sites for different transcription factors on the predicted TFBS cluster in the gene *Hairy* for *D*. *melanogaster*, C) Locations of the gene, TSS and the TFBS cluster as predicted by the MCAST software for the *Hairy* gene in *T*. *castaneum*, D) Binding sites for different transcription factors on the predicted TFBS cluster in the gene *Hairy* for *T*. *castaneum*. 4.3 shows the predicted transcription factor binding site clusters in *Runt* for *D*. *melanogaster* and *T*. *castaneum*. A) Locations of the gene, TSS and the TFBS cluster as predicted by the MCAST software for the *Runt* gene in *D*. *melanogaster*, B) Binding sites for different transcription factors on the predicted TFBS cluster in the gene *Runt* for *D*. *melanogaster*, C) Locations of the gene, TSS and the TFBS cluster as predicted by the MCAST software for the *Runt* gene in *T*. *castaneum*, D) Binding sites for different transcription factors on the predicted TFBS cluster in the gene *Runt* for *T*. *castaneum*.

Fig 4 shows the results for the TFBS clusters as predicted by the MCAST software for the *Even-skipped*, *Hairy*, and *Runt* pair-rule genes in *D*. *melanogaster* and *T*. *castaneum*. The cluster of TFBS in both *Even-skipped* and *Runt* genes are present upstream of the TSS in both *D*. *melanogaster* and *T*. *castaneum* (Fig 4.1 and 4.3). Fig 4.2, shows the cluster of TFBS on the *Hairy* gene. The cluster is present upstream to the TSS in *D*. *melanogaster* and downstream to the TSS in *T*. *castaneum*.

**Fig 5:**
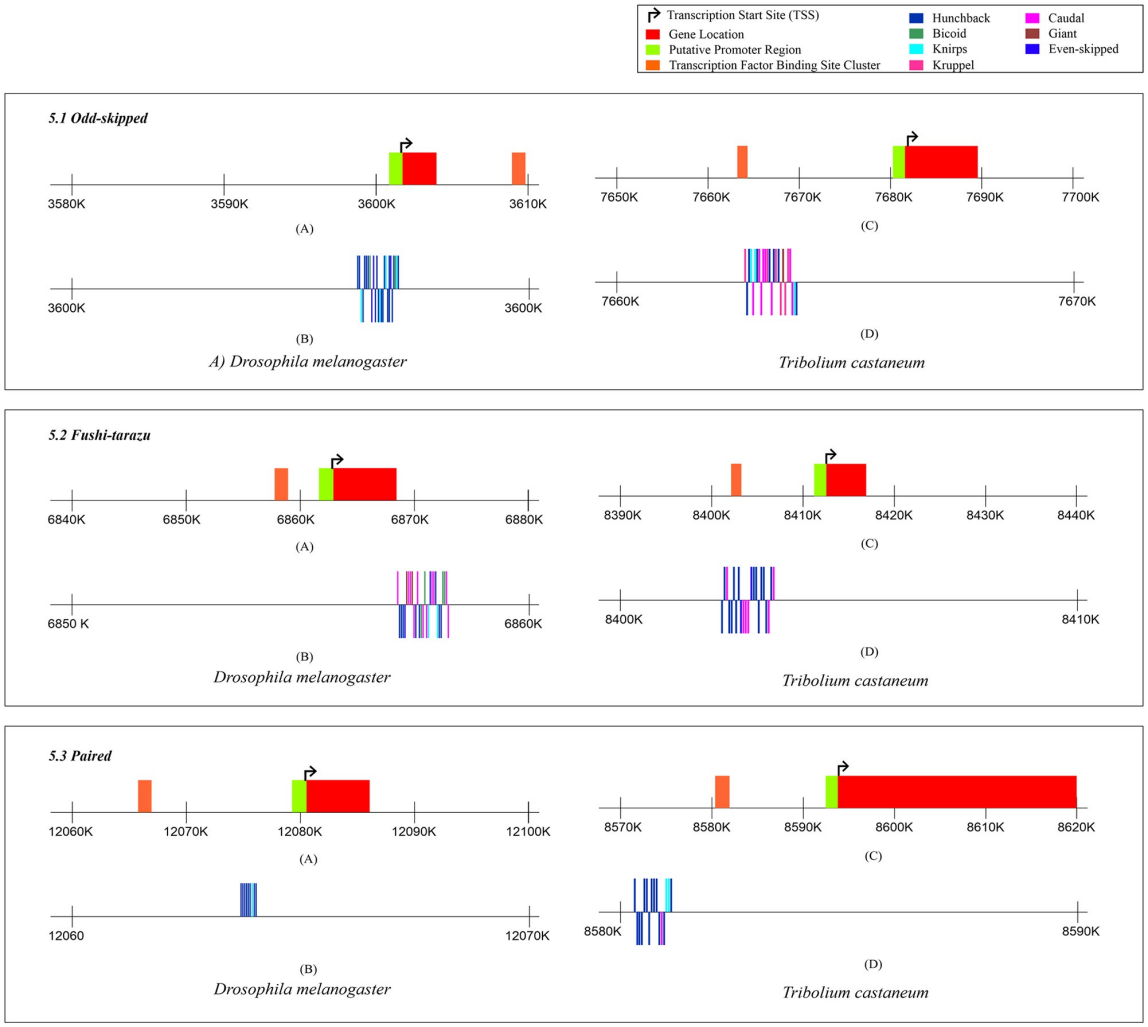
Figure showing the pCRM in *Drosophila melanogaster* and *Tribolium castaneum* for different transcription factors in *Odd-skipped*, *Fushi-tarazu*, and *Paired* genes. 5.1 shows the predicted transcription factor binding site clusters in *Odd-skipped* for *D*. *melanogaster* and *T*. *castaneum*, A) Locations of the gene, TSS and the TFBS cluster as predicted by the MCAST software for the *Odd-skipped* gene in *D*. *melanogaster*, B) Binding sites for different transcription factors on the predicted TFBS cluster in the gene *Odd-skipped* for *D*. *melanogaster*, C) Locations of the gene, TSS and the TFBS cluster as predicted by the MCAST software for the *Odd-skipped* gene in *T*. *castaneum*, D) Binding sites for different transcription factors on the predicted TFBS cluster in the gene *Odd-skipped* for *T*. *castaneum*. 5.2 shows the predicted transcription factor binding site clusters in *Fushi-Tarazu* for *D*. *melanogaster* and *T*. *castaneum*. A) Locations of the gene, TSS and the TFBS cluster as predicted by the MCAST software for the *Fushi-Tarazu* gene in *D*. *melanogaster*, B) Binding sites for different transcription factors on the predicted TFBS cluster in the gene *Fushi-Tarazu* for *D*. *melanogaster*, C) Locations of the gene, TSS and the TFBS cluster as predicted by the MCAST software for the *Fushi-Tarazu* gene in *T*. *castaneum*, D) Binding sites for different transcription factors on the predicted TFBS cluster in the gene *Fushi-Tarazu* for *T*. *castaneum*. 5.3 shows the predicted transcription factor binding site clusters in *Paired* for *D*. *melanogaster* and *T*. *castaneum*. A) Locations of the gene, TSS and the TFBS cluster as predicted by the MCAST software for the *Paired* gene in *D*. *melanogaster*, B) Binding sites for different transcription factors on the predicted TFBS cluster in the gene *Paired* for *D*. *melanogaster*, C) Locations of the gene, TSS and the TFBS cluster as predicted by the MCAST software for the *Paired* gene in *T*. *castaneum*, D) Binding sites for different transcription factors on the predicted TFBS cluster in the gene *Paired* for *T*. *castaneum*.

The CRMs for *Odd-skipped*, *Fushi-tarazu*, and *Paired* genes as predicted by the MCAST software are depicted in Fig 5. Fig 5.1 exhibits the cluster of TFBS on the *Odd-skipped* gene. The cluster of is present downstream to the TSS in *D*. *melanogaster* and upstream in *T*. *castaneum*. The clusters of TFBS in both *Fushi-tarazu* and *Paired* genes are present upstream to the TSS in both the insect species (Fig 5.2 and 5.3).

The results depicted by the MCAST software, that is, location of the TSS, TFBS cluster, and size of the putative cluster in base pairs are summarized in Table 3 for *D*. *melanogaster* and Table 4 for *T*. *castaneum*. Table 5 depicts the number of transcription factor binding sites in all genes predicted by the software in both the insect species.

**Table 3:** Location of the transcription start sites and putative cis-regulatory modules and CRM’s size as predicted by the MCAST software on the A-P patterning genes of *Drosophila melanogaster*. Here–and *+* represents the upstream and downstream location of predicted CRM respectively.

S. No.	Gene Name	Transcription Start Site	Predicted CRM’s location (motif score of pCRM)	Predicted CRM’s size	+,- location of pCRM
1	*Hunchback*	8690979	8684092–8684568 (64.46)	476 bp	-
2	*Knirps*	20692326	20694580–20695169 (84.08)	589 bp	-
3	*Caudal*	20770700	20777,675–20778,195 (28.18)	520 bp	+
4	*Kruppel*	25226609	25222746–25223355 (59.56)	609 bp	+
5	*Giant*	2427112	2412138–2412768 (44.80)	630 bp	-
6	*Tailless*	30852314	30842160–30842579 (44.68)	419 bp	-
7	*Even-skipped*	9979430	9974834–9975399 (42.35)	565 bp	-
8	*Odd-skipped*	3604995	3609367–3609952 (59.35)	585 bp	+
9	*Runt*	20694432	20685463–20686280 (73.21)	817 bp	-
10	*Hairy*	8675953	8665577–8666104 (85.88)	527 bp	-
11	*Fushi-tarazu*	6864324	6858324–6858811 (45.67)	487 bp	-
12	*Paired*	12082992	12067972–12068189 (18.52)	217 bp	-

**Table 4:** Location of the transcription start sites and putative cis-regulatory modules and predicted CRM’s size as predicted by the MCAST software on the A-P patterning genes of *Tribolium castaneum*.

S.No.	Gene Name	Transcription Start Site	Predicted CRM’S location (motif score of pCRM)	Predicted CRM’S size	+,- location of pCRM
1	*Hunchback*	8102130	8092849–8093278 (11.55)	429 bp	-
2	*Knirps*	4721907	4697483–4698242 (23.50)	759 bp	-
3	*Caudal*	2828217	2832700–2833237 (12.13)	537 bp	+
4	*Kruppel*	6495152	6502223–6502912 (23.50)	689 bp	+
5	*Giant*	3766076	3765042–3765574 (19.20)	532 bp	-
6	*Tailless*	11221703	11230486–11230843 (15.45)	357 bp	+
7	*Even-skipped*	624731	617466–617724 (19.58)	258 bp	-
8	*Odd-skipped*	7673308	7667718–7668180 (46.94)	462 bp	-
9	*Runt*	11253714	11243118–11243747 (18.58)	629 bp	-
10	*Hairy*	15627964	15635988–15636522 (26.15)	534 bp	+
11	*Fushi-tarazu*	8414663	8404780–8405724 (20.87)	944 bp	-
12	*Paired*	8598708	8580847–8581470 (28.38)	623 bp	-

**Table 5:** Number of Transcription Factor binding sites on different A-P patterning genes in *Drosophila melanogaster* and *Tribolium castaneum* as predicted by the MCAST software. Here, *Drosophila melanogaster* and *Tribolium castaneum* are abbreviated as *Dm* and *Tc* respectively.

S.No.	Name of the A-P patterning gene	Transctription Factors													
		Bicoid		Hunchback		Caudal		Giant		Knirps		Kruppel		Even-skipped	
		*Dm*	*Tc*	*Dm*	*Tc*	*Dm*	*Tc*	*Dm*	*Tc*	*Dm*	*Tc*	*Dm*	*Tc*	*Dm*	*Tc*
1	***Hunchback***	6	-	10	5	-	4	3	-	4	-	1	-	-	-
2	***Knirps***	4	-	17	9	-	1	-	-	3	-	3	-	-	-
3	***Caudal***	8	-	9	8	6	3	-	-	-	-	-	-	-	-
4	***Kruppel***	6	-	11	11	-	3	2	-	7	-	6	-	-	-
5	***Giant***	2	-	9	5	1	5	1	-	3	-	9	1	3	-
6	***Tailless***	-	-	6	6	6	3	-	-	6	-	-	-	-	-
7	***Even-skipped***	-	-	9	10	-	-	-	-	7	3	-	-	-	-
8	***Hairy***	-	-	14	11	5	2	1	1	10	-	6	-	-	-
9	***Runt***	-	-	17	11	4	3	1	1	8	1	-	-	-	-
10	***Odd-skipped***	2	-	17	8	-	7	-	1	4	3	-	6	3	-
11	***Fushi-tarazu***	4	-	8	13	11	6	2	-	2	-	-	-	2	3
12	***Paired***	-	-	8	13	-	1	-	-	1	2	-	-	-	-

### Multiple sequence alignment, conserved sites, transitional pairs, transversional pairs, and transition/transversion rate

All the predicted pCRMs were subjected to multiple sequence alignment using the CLUSTALW [30] tool in Bioedit software [31]. These were further analysed for the presence of conserved sites, transition pairs, transversional pairs, and transition/transversion rate using MEGA XI software [32]. The results for the analysis are depicted in Table 6.

**Table 6:** Number of the conserved, transition and transversional sites along with the transition/transversion ratio in the predicted pCRMs as depicted by the MEGA XI software.

S.NO.	Name of Gene	Number of conserved sites	Number of transition sites	Number of transversional sites	Transition/Transversional ratio
***1***	*Hunchback*	216	71	79	0.9
***2***	*Knirps*	267	134	178	0.8
***3***	*Caudal*	214	125	168	0.7
***4***	*Kruppel*	276	160	167	1.0
***5***	*Giant*	234	133	162	0.8
***6***	*Tailless*	134	80	111	0.7
***7***	*Even-skipped*	128	53	74	0.7
***8***	*Odd-skipped*	221	102	131	0.8
***9***	*Runt*	268	153	200	0.8
***10***	*Hairy*	201	133	184	0.7
***11***	*Fushi-tarazu*	271	78	137	0.6
***12***	*Paired*	84	31	102	0.3

### Interaction between the TFs

**Fig 6:**
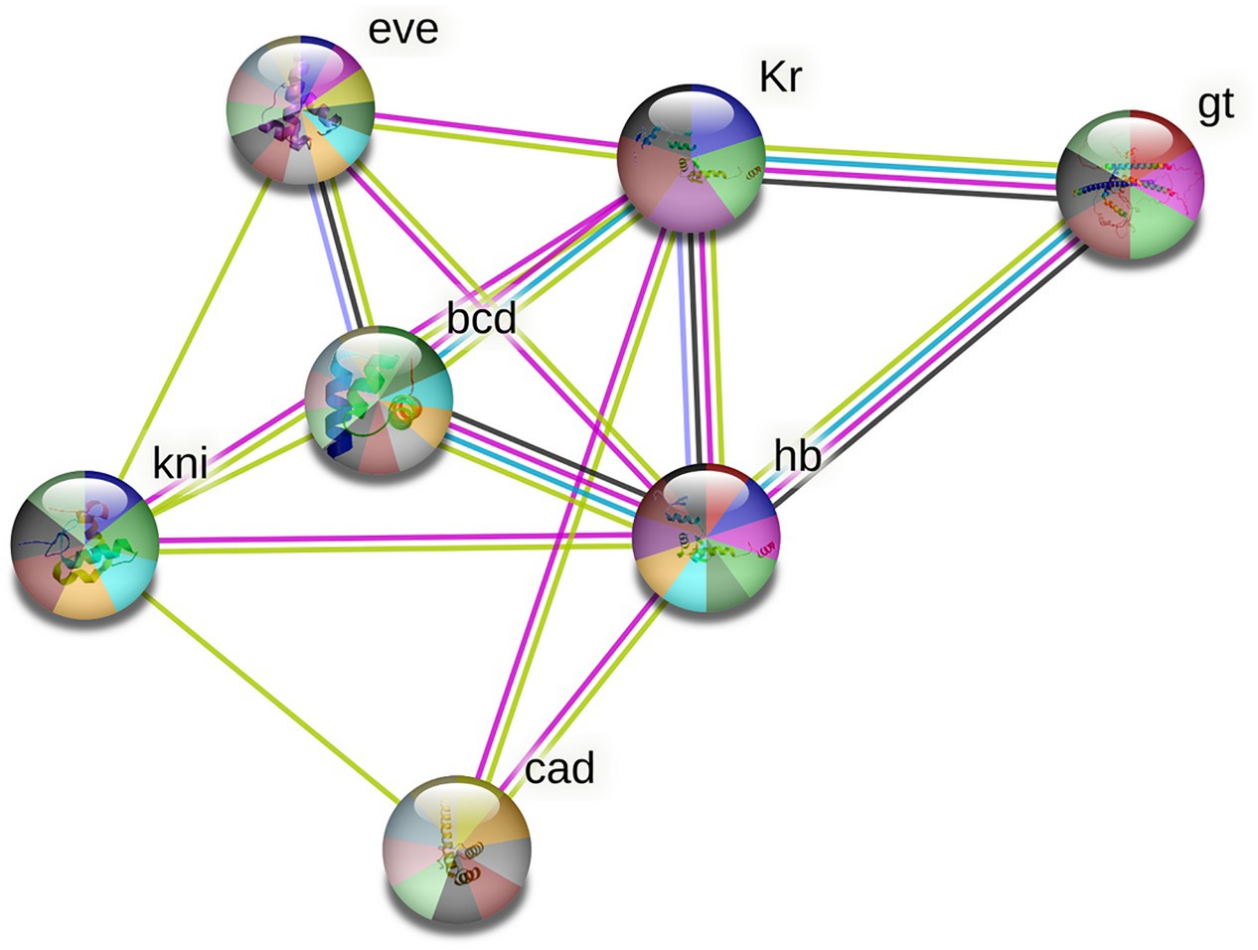
Protein-protein interactions between the different TFs which bind to the enhancers in the A-P patterning genes of *D*. *melanogaster*. Here in this figure, the pink colour represents the experimentally determined interactions, Sky blue shows the interactions curated from databases between the proteins. The blue colour node represents the gene co-occurrence. The black colour node depicts the co-expression of protein and the violet colour represents the protein homology. The yellow colour node represents the interactions that are predicted through text mining.

**Fig 7:**
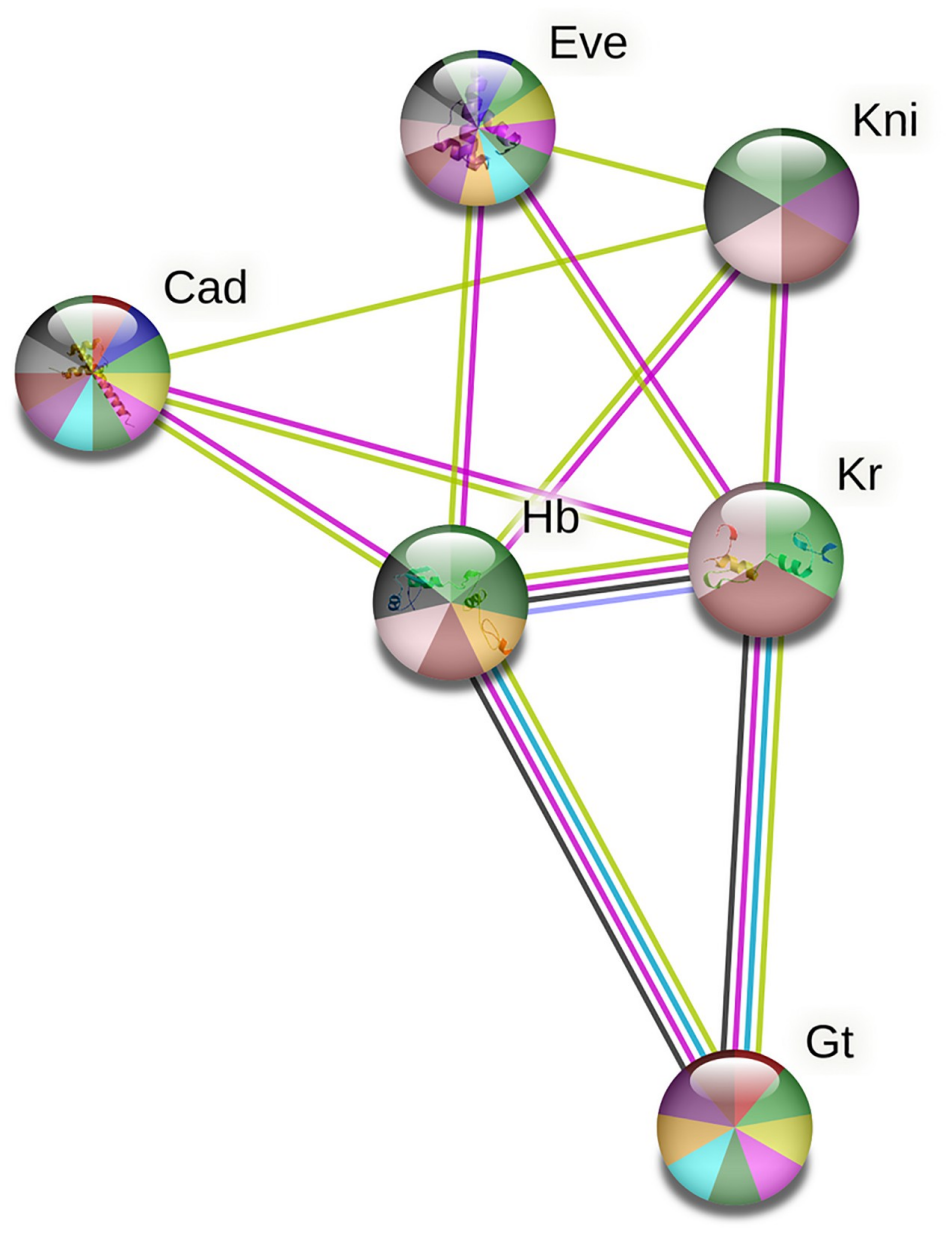
Protein-protein interactions between the different TFs which bind to the enhancers in the A-P patterning genes of *T*. *castaneum*. Here in this figure, the pink colour represents the experimentally determined interactions, Sky blue shows the interactions curated from databases between the proteins. The blue colour node represents the gene co-occurrence. The black colour node depicts the co-expression of protein and the violet colour represents the protein homology. The yellow colour node represents the interactions that are predicted through text mining.

Figs 6 and 7 depict the interactions between Bicoid, Hunchback, Caudal, Knirps, Kruppel, Giant, and Even-skipped transcription factors of *D*. *melanogaster* and *T*. *castaneum* respectively. These interactions were evaluated using the STRING database. As predicted by the software, the average local clustering coefficient is 0.857 for *D*. *melanogaster and* 0.837 for *T*. *castaneum*. The PPI enrichment p-value is < 1.0e-16 for *D*. *melanogaster* and 3.28e-12 for *T*. *castaneum*. The interaction between the TFs in both *D*. *melanogaster* and *T*. *castaneum* shows more enrichment, which suggested that these TFs proteins are biologically connected as a group.

In *D*. *melanogaster*, the software predicted that the transcription factor bicoid shows co-expression with Hunchback. Hunchback shows protein homology with Kruppel. There was no co-expression of proteins detected in the *T*. *castaneum* and protein homology was identified between Hunchback and Kruppel as in *D*. *melanogaster*. The software depicted known experimentally determined interactions between all proteins except for Caudal, Knirps, and Even-skipped in both *D*. *melanogaster* and *T*. *castaneum*. No interaction between Caudal and Even-skipped proteins were predicted in both insects.

## Discussion

The determination of the early body design in *D*. *melanogaster* is done by the action of cis-regulatory elements in the genome that regulate gene expression during development. Transcription factors (TFs) bind to these regions and regulate target gene expression [34]. Translation of localised maternal mRNAs during oogenesis constructs the initial TF gradients in the embryo at the top of the A-P patterning cascade [35, 36]. These maternal TFs then bind to gap gene specific embryonic regulatory regions, thereby regulating gap TF expression patterns during early A-P specification [9, 37]. The interaction between the different TFs was predicted using the STRING database. Based on computational modelling, it seems that these TFs are interconnected and have a role in coordinating the insect development. Previous studies suggested that the self-regulation pathway and the activities of other maternal-effect genes and gap genes control the expression of gap genes involved in the establishment of the A-P axis. [21]. In addition to the regulation of gap genes, these genes act as TFs for the regulation of genes involved in the A-P axis formation cascade which are pair-rule genes, segment polarity genes, and Homeotic selector genes [21, 38]. Consistent with the interaction identified by STRING software in the present study, previous studies on Bicoid and Hunchback have revealed that these proteins have compatible binding with each other [39]. The Kruppel protein interacts with the Hunchback protein in order to repress the latter’s expression and simultaneously regulate its own expression, as evidenced by previous research [40]. The findings of our study indicate that the proteins Hunchback and Kruppel exhibit homology, implying a shared evolutionary ancestry between these proteins. Gap TFs also control the downstream cascade of A-P Patterning genes [41, 42]. The binding of TFs to particular clusters of activator and repressor binding sites inside embryonic CRMs tightly controls gene expression patterns at each phase in the cascade. Individual CRMs have distinct molecular characteristics that influence transcriptional output. When a TF attaches to a CRM, it might behave as an activator or a repressor, depending on the situation [43, 44]. Hundreds of cis-regulatory motif sequences have been identified across all model species using both experimental [45–48] and bioinformatic [49–52] approaches, as well as the discovery of the related transcription factors binding to them [53]. The cluster of these transcription factor binding sites can be recognized as putative enhancers or CRM which help in the process of transcription. Many key regulators of early development have been identified courtesy of sophisticated genetic screening and the molecular biology and biochemistry of these factors, as well as their target sequences, have gained considerable interest in *Drosophila* [54, 55]. In-silico identification of CRM in early development in *D*. *melanogaster* and *D*. *pseudoobscura* were predicted and in-vivo testing was done on pCRMs [1, 56]. *Tribolium* is a good illustration of short germ embryogenesis in insects since it represents the ancestral kind of embryogenesis. In comparison to *Drosophila*, the blastoderm phase determines only the cephalic and thoracic segments, but not the abdominal segments. While the Bicoid gradient has been used to study pattern development in *Drosophila*, it is considered that this is not the case in *Tribolium* as *Bicoid* is not present in the insect. The alteration from C*audal* activation of the *Hunchback* gap region to direct activation by *Bicoid* was an evolutionary shift from short to long germ embryogenesis [38, 57]. The previous studies on A-P patterning comparison have been done on different species of *Drosophila* but there is no study which compares A-P patterning in different insect species belonging to different insect order [58]. Hence, the present study was performed to check whether the insects belonging to different orders, have different developmental patterns but have similar genes, and are controlled by similar cis-regulatory modules or not.

In *D*. *melanogaster*, the zygotic *Hunchback* gene is activated by the synergetic interaction between the Hunchback and Bicoid transcription factors [59–61], the result predicted by the present study also depicted a similar result. The cluster predicted by MCAST has the maximum number of Hunchback transcription factor sites in both *D*. *melanogaster* and *T*. *castaneum*. As mentioned above, in *Tribolium* the *bicoid* gene is absent therefore *Tribolium* has additional *caudal* sites which help in *Hunchback* gene activation and expression. In the case of *Knirps*, the maximum number of transcription factors binding sites again are of *Hunchback* in addition to other factors in both *D*. *melanogaster* and *T*. *castaneum*. Previous *Drosophila* studies suggest that the *Hunchback* acts as a repressor for *Knirps*, as binding of the *Hunchback* suppresses the *Knirps* in the anterior half of the embryo [62, 63] *Caudal* gene is one of the most studied gap genes in *D*. *melanogaster* and *T*. *castaneum*, which help in the activation of gap genes and pair-rule genes in insects. *Caudal* is known to be a downstream core promoter element [39] and our results also depict the same. Both in *D*. *melanogaster* and *T*. *castaneum* the cluster is found to be downstream to the transcription start site. Both clusters have TFBS for Hunchback and Caudal, while *Drosophila* has additional sites for Bicoid also.

The previous studies of the Kruppel gene suggest that the *Kruppel* has TFBS for Hunchback, Bicoid, Giant, and Knirps [62, 64]. *Hunchback* and *Bicoid* are known as activators of the *Kruppel* gene, while *Knirps* and *Giant are* known to be their repressors [62]. The present study result has also predicted similar binding sites in *D*. *melanogaster* as in evident in previous studies while *T*. *castaneum* has TFBS clusters for Hunchback and Caudal only. For the expression of *Giant* gene in *Drosophila*, Hunchback functions as a concentration-dependent repressor of *Giant*, suppressing its most anterior expression [65–67]. Giant will not be transcribed in the anterior domain without the presence of *Bicoid* [65]. Activation in the posterior domain necessitates the combined actions of the *Caudal* and *Bicoid* [65, 68]. Similar TFBS was found in *D*. *melanogaster* and *T*. *castaneum*. In addition to these predicted pCRMs, binding sites for Kruppel and Knirps were predicted in *D*. *melanogaster* while only one Kruppel site was predicted in *T*. *castaneum*. These results suggest that the gap genes transcription in *D*. *melanogaster* is mainly controlled by Hunchback, Bicoid, and Caudal proteins while in *T*. *castaneum*, Hunchback and Caudal are majorly the transcription factors for the gap genes.

Gap genes with maternal gradients act across shorter distances and overlap at their borders, resulting in the seven-stripe expression patterns of the pair-rule genes [69, 70] *Even-skipped* is one of the most extensively studied pair-rule gene in *D*. *melanogaster*. Previous studies documented the individual *Even-skipped* stripe responds to the different gradients and combinations of gap gene transcription factors [43, 71–73]. The present study predicts the gene has TFBS clusters for Hunchback and Knirps only in both *D*. *melanogaster* and *T*. *castaneum*, this combination in previous studies was found to influence the stripe 3+7 enhancer in *Drosophila melanogaster* [74]. In the earlier *D*. *melanogaster* study, it was found that the gap genes mainly *Hunchback*, *Giant*, *Kruppel*, and *Knirps* act as a repressor for the *Runt*, which is a primary pair-rule gene [75]. Similar TFBS were predicted in the present study, with the exception that no Kruppel binding site was found in both *D*. *melanogaster* and *T*. *castaneum*. In addition to these known TFs, additional binding sites for Caudal were also found in both *D*. *melanogaster* and *T*. *castaneum*. The gap genes mainly, *Hunchback*, *Knirps*, and *Kruppel* are known to influence the expression of pair-rule genes [76, 77]. The *hairy* gene also has similar TFBS, which is alike to the cluster predicted in *D*. *melanogaster*. The cluster have TFBS for Hunchback, Knirps, and Kruppel in addition to these, binding sites for Caudal and one Giant transcription factor were also predicted. In contrast to *D*. *melanogaster*, hairy gene in *T*. *castaneum* showed binding sites for Hunchback, Caudal and Giant site. The *Hairy* functions in the trunk and head segmentation in *D*. *melanogaster*. A previous study on *Hairy* gene in *T*. *castaneum* suggests that the gene functions only during trunk segmentation in *T*. *castaneum* and is non-functional during the head segmentation pathway [78]. The *Fushi-tarazu*, *Odd-skipped*, and *Paired* are regarded as secondary pair rule genes in *D*. *melanogaster* [3, 79]. These genes are known to have Hunchback Kruppel, Knirps and Giant as repressor factors in different combinations [80]. The MCAST analysis revealed binding sites for Hunchback, Caudal and Even-skipped factors in the secondary pair rule genes for both *D*. *melanogaster* and *T*. *castaneum*. In addition, *Drosophila Fushi-tarazu* gene showed binding sites for bicoid and giant also. As far as the *Odd skipped* gene is concerned, both *D*. *melanogaster* and *T*. *castaneum* have TFBS for Hunchback and Knirps. The gene in *D*. *melanogaster* also showed binding sites for Bicoid and Even-skipped. The *Odd skipped* gene in *T*. *castaneum* showed binding sites for Kruppel and Caudal also. *Paired* being a secondary pair-rule gene, is controlled by the primary pair-rule genes in *D*. *melanogaster* and *T*. *castaneum* [81]. Most of the Predicted Cis-regulatory elements of the pair rule genes have TFBS for the Hunchback, Bicoid, Caudal, Knirps, and Kruppel in *D*. *melanogaster*. However, in *T*. *castaneum*, most pCRMs have TFBS for hunchback, knirps, caudal, and kruppel. The size of the predicted Cis-regulatory elements is between 200 bp to 850 bp for *D*. *melanogaster* and 240 bp– 950 bp for *T*. *castaneum*. The results of the MCAST analysis in the present study suggest that most of the transcription factors which control the A-P patterning cascade are conserved in *D*. *melanogaster* and *T*. *castaneum* with the exception that there are no binding sites for Bicoid in *T*. *castaneum*.

Transitions (Ti) are referred to as pyrimidine- or purine-based A-G or C-T switching. A transversion (Tv) is the exchange of two-ring purine nucleobases for one-ring pyrimidine bases. There are four conversion possibilities: A-C, A-T, C-G, and G-T. From the last decade, the Ti/Tv ratio has been employed as a significant metric for the reconstruction of phylogenetic trees and the calculation of divergence. Even the High-throughput sequencing studies employ the Ti/Tv ratio as a quality control measure. Assuming that there are two potential transitions and four possible transversions, the Ti/Tv ratio, which divides the number of transition SNPs by the number of transversion SNPs, should equal 0.5 if replacement variations occur at random. Nonetheless, a transversion is considered a more important change than a transition since it requires more energy than replacement without affecting the ring structure. Hence, the transition and transversion ratio is frequently more than 0.5 in actual sequencing data [82–85]. Studies also suggest that the Tv’s have more significant effects on regulatory DNA, such as TF binding motif studies and allele-specific TF binding [86]. Keeping in mind, the importance of Ttransitions and transversion, the present study also evaluated the number of Ti, Tv sites, and Ti/Tv ratio in the predicted enhancers of A-P patterning genes. The Tv sites were found to be more than the Ti sites and the average Ti/Tv ratio was 0.75 as given in Table 5. As the Tv is most likely to affect the amino acid sequence than the Ti, the more Tv can be indicative of a large number of variations, which will ultimately affect gene expression [81–85].

## Conclusion

This study marks the first-ever attempt to conduct an integrated examination of the location, size, and composition of clusters of transcription factor binding sites (TFBS) within the cis-regulatory elements of multiple anterior-posterior (A-P) patterning genes that exhibit orthology to gene sequences found in *Drosophila*. The present investigation has revealed that comparable transcription factors (TFs) could regulate the expression of anterior-posterior (A-P) patterning genes in Diptera and Coleoptera taxa of insects. The majority of transcription factors (TFs) were observed to be situated upstream of the transcription start site (TSS), although a subset were also identified downstream of the TSS. In both *Drosophila melanogaster* and *Tribolium castaneum*, the Hunchback transcription factor binding site (TFBS) exhibited the highest frequency among all identified TFBS. The present study contributes to the advancement of our understanding regarding the evolutionary patterns of genes and cis-regulatory elements in two distinct orders of insects. Subsequent validation of these findings may be achieved through in-vitro and in-vivo experimentation.
